# Participants’ Experiences with a Telerehabilitation Behavioral Exercise Intervention to Improve Physical Function and Physical Activity After Lung Transplantation: A Multi-Method Study

**DOI:** 10.63144/ijt.2025.6705

**Published:** 2025-06-12

**Authors:** Sweta Jena, Elizabeth Tarazi, Bryan Willey, Andrea Hergenroeder, Christopher Imes, Elizabeth Lenderman, Seol Ju E. Moon, Kristen Jones, Melissa Vendetti, Annette DeVito Dabbs

**Affiliations:** 1 University of Pittsburgh, School of Nursing, Pittsburgh, PA, USA; 2 University of Pittsburgh, School of Health and Rehabilitation Sciences, Pittsburgh, PA, USA; 3 University of Minnesota, School of Nursing, Minneapolis, MN, USA

**Keywords:** Lung transplantation, Telerehabilitation

## Abstract

Few studies have examined the factors influencing participant acceptability and engagement of telerehabilitation-based interventions. The study purpose was to describe the experiences of participants who were randomly assigned to the Lung Transplant Go (LTGO) telerehabilitation intervention group of a two-group, randomized, controlled parent trial. Survey measures included usability and familiarity with technology, exercise self-efficacy, and intervention adherence rates. Semistructured interviews (SSI) were completed at 3- and 6-months post-intervention. Survey scores and adherence rates were high. SSI responses were generally positive with reported benefits including motivation, building strength, and becoming more physically active. Frustrations reported with technology or delivery were few and promptly resolved. Participants were willing to participate in the LTGO intervention again. Those who demonstrate limited familiarity with information technologies may benefit from additional support prior to intervention delivery.

Lung transplantation is an established treatment option for persons with advanced lung disease to regain quality of life and longevity (Perch et al., 2022; Valapour et al., 2023). Prior to transplant, respiratory limitations reduce ventilatory capacity, leading to disabling dyspnea and fatigue, which restrict physical activity and exercise tolerance. After transplant, lung function typically returns to near normal levels, and lung transplant recipients (LTR) report improvements in quality of life compared with pretransplant values (Kolaitis & Singer, 2018). However, exercise capacity remains low due to chronic deconditioning which undermines the intended benefits of the highly selective, resource-intensive transplant procedure (Kobashigawa et al., 20196). Overwhelming evidence supports the benefits of pulmonary rehabilitation (PR) to improve functional exercise capacity in persons with end-stage lung disease, including LTR (Rochester et al., 2015; Wickerson et al., 2015). Therefore, PR is prescribed after lung transplantation to optimize functional status (Abidi et al., 2023; Gutierrez-Arias et al., 2021).

Yet, despite its potential benefits, due to multiple barriers, uptake and completion rates of PR are alarmingly low (Arkhipova-Jenkins & Helfand, 2020; Lahham & Holland, 2021). Barriers include lack of access to local PR programs, low start and completion rates (especially for people living in rural areas with limited incomes who lack insurance coverage for PR), lack of transportation, scheduling issues, or disruption of usual routines (Fischer et al., 2009; Spruit et al., 2014; Jones et al., 2017). Moreover, during in-person PR, exercise occurs under close clinician supervision but, afterward, LTR are expected to continue exercising independently at home while many do not feel confident or motivated to exercise alone (Selzer et al., 2016; Levack et al., 2018). Importantly, while behavior change strategies essential to achieving exercise goals, sustaining exercise, and maintaining an active lifestyle, are included in guidelines for PR programs, implementation of these change strategies may vary widely. The delivery and receipt of specific behavior change strategies, such as goal setting, motivation building, self-monitoring and promotion of exercise self-efficacy among others included in this study protocol, may not always be documented or considered in the analysis of impact of traditional in-person PR programs. Consequently, LTR may have little opportunity to establish behavior patterns that promote continued exercise self-management.

We posit that characteristics inherent to in-person PR programs limit the initiation and long-term adherence of LTR to exercise. Studies of remote options for delivering PR have shown promise (Chan et al., 2016; Holland et al., 2021; Singer et al., 2018), but the trials to date have been small, retrospective, or lacked comparison groups (Bonnevie et al., 2021; Myers et al., 2021). Furthermore, the active intervention periods were brief (3 months) and did not include a maintenance phase or the behavioral change strategies that are known to help sustain the benefits of a formal exercise training program.

Telerehabilitation is defined as delivering rehabilitation at a distance using various interactive telehealth technologies, (e.g., live video conferences, mobile apps, automated chatbots) (Fekete et al., 2021; Peretti et al., 2017). Many studies suggest that telerehabilitation is cost-effective and associated with participant satisfaction and adherence, presumably because it reduces many of the aforementioned barriers to in-person PR (Choi et al., 2016; Dennett et al., 2021; Hansen et al., 2020; Subedi et al., 2020). Thus, telerehabilitation is a promising option for promoting exercise after lung transplant. The LTGO telerehabilitation intervention was designed to offer an effective and acceptable alternative to in-person PR.

It is increasingly acknowledged that the participants’ experiences should be considered when evaluating interventions (Sekhon et al., 2017). Health intervention studies are leveraging interactive health technologies such as telerehabilitation (Schein, Schmeler, Saptono et al., 2010), yet little empirical data about factors influencing acceptability and engagement of behavioral interventions exist (Materia & Smyth, 2021). Assessments of acceptability of health technologies will help determine how well an intervention was received by the target population and the extent to which the intervention or its components met their needs (Ayala & Elder, 2011). Therefore, the purposes of this study were to describe: (1) the experiences of LTR assigned to the LTGO telerehabilitation behavioral exercise intervention arm of a randomized controlled trial (RCT) and (2) the impact of factors thought to influence the participants’ subjective experiences with the telerehabilitation intervention.

## Methods

### Design

The study used an exploratory, multi-method approach (Fetters et al., 2013) to characterize the experiences of participants randomized to the LTGO intervention by integrating quantitative data from a battery of surveys, degree of adherence to LTGO exercise training, behavior coaching sessions, and monthly phone calls completed over a 6-month study period with the qualitative findings from the semi-structured interviews (SSI) at 3- and 6-months post-intervention.

### Sample and Setting

Participants of the parent RCT were adult LTR at one of the world’s largest lung transplant programs affiliated with an academic tertiary medical center in the Mid-Atlantic region of the U.S. The study was approved by an institutional review board, and all participants provided informed consent. Inclusion criteria included: between 1–18 months post-transplant, discharged to home, and self-reported difficulty walking ¼ mile or climbing 10 steps without resting. Exclusion criteria included conditions that precluded exercise, no home internet, and concurrent participation in a formal pulmonary rehab program (Vendetti et al., 2023). After the onset of COVID in March of 2020, all study activities, including recruitment, informed consent, intervention delivery and outcome assessments were completed remotely with participants in their homes using a 2-way, Health Insurance Portability and Accountability Act compliant videoconference platform (Moon et al., 2021). All parent trial participants received clinical management according to the transplant program’s standard protocol, including a referral to PR. After completion of the baseline assessment, participants were randomized 1:1 to either the LTGO telerehabilitation group or enhanced usual care (EUC) group (self-directed activity tracking and receiving monthly newsletters) by the trial statistician (DR).

Forty-four LTR were randomized to the LTGO intervention. The sample for this analysis included only the 38/44 LTR who completed the SSI. Sample characteristics are reported in Table 1. The mean age was 57.9 years (SD= 11.9) and 34 (82%) of participants were White; 21 (55%) were male. Of all the participants, 20 (52%) reported receiving some college education. Most were married or living with a partner 27 (71%), unemployed or disabled 33 (86%), with 32 (84%) reporting incomes that met their needs.

### The LTGO Telerehabilitation Intervention

The intervention was designed by experts in exercise for persons with chronic cardiopulmonary conditions (AH, BW) and delivered by the interventionist (BW) in two-phases over a six-month period (Hergendroeder et al., 2023). Phase 1 was comprised of 12-weekly, intensive, supervised home-based exercise training and behavioral coaching sessions via a videoconferencing platform using a Health Insurance Portability and Accountability Act (HIPAA)-sponsored Zoom account that provided privacy and security safeguards for the sessions (Moon et al., 2021). Participants used a laptop or tablet at home with a functioning video camera and speakers. All sessions were supervised by the interventionist and included exercises and instructions for physical activity, progressive resistance training, flexibility, and balance exercises. A total of eight brief behavioral coaching lessons were integrated into the sessions to help the participants develop the skills to self-manage physical activity and maintain this behavior as a sustained habit. The topics included how to set SMART goals, monitor exercise, the Frequency, Intensity, Time, and Type (FITT) principles of exercise prescription, breathing techniques, time management, setbacks and barriers, social support, and stress management. Phase 2, the transition to exercise self-management phase, was comprised of a behavioral contract followed by three monthly telephone sessions. The interventionist contacted participants by phone to provide behavioral coaching and exercise reinforcement.

### Survey Measures

All surveys were administered by assessors blinded to group assignment (CI, SJEM, KJ). The LTR completed a sociodemographic survey, and other standardized measures that were selected based on their potential to influence the participants’ subjective experience with a telerehabilitation behavioral exercise intervention (full surveys included in the Appendices).

#### Information Technology Familiarity Questionnaire (ITFQ)

The ITFQ (Faett, et al., 2013), a measure of a person’s familiarity with information technology, was included to assess a potential barrier to success and satisfaction using a telerehabilitation intervention. The ITFQ was completed by participants before the LTGO intervention. The tool consisted of eight questions on frequency of use of information technology which participants rated using a Likert scale (1= daily use to 3= never used). A lower score indicates a higher level of familiarity. The total score is the sum of the scores of the eight questions, ranging from 8–24. The face validity of the ITFQ was evaluated by researchers with experience conducting telerehabilitation and found to be acceptable (Faett et al., 2013). See Appendix A.

#### Telerehabilitation Usability Questionnaire (TUQ)

The TUQ is a validated self-report measure of usability for a variety of telerehabilitation systems (Faett et al., 2013; Parmanto et al., 2013; Schein, Schmeler, Holm et al., 2010). The TUQ was administered after the completion of phase 1 to assess the usability of the two-way video communication system. Participants were asked to rate the degree to which they agreed with 21 statements that assessed six components of usability: usefulness, ease of use, interface quality, interaction quality, reliability, and satisfaction on a Likert scale (1 = disagree through 7 = agree). A higher score indicates higher acceptability. The total score is computed by determining a mean score for the 21 questions with a total possible score of 7. See Appendix B.

#### Self-Efficacy to Regulate Exercise (SERE)

Perceived self-efficacy is the degree to which a person believes in their capacity to achieve given goals (Bandura, 1997). The SERE is a self-report measure of perceived capacity to perform an exercise routine regularly, defined in the survey as three or more times a week. The SERE includes 18 items for participants to rate the strength of their belief in their ability to execute the requisite activities under certain situations on a 100-point scale, ranging in 10-unit intervals from 0 (“Cannot do”); through intermediate degrees of assurance, 50 (“Moderately certain can do”); to complete assurance, 100 (“Highly certain can do”). Ratings are summed and an average SERE score is calculated. The SERE was deemed reliable and valid in measuring significant improvements in a wide range of regulatory behaviors and populations (Oaten & Cheng, 2010; Garcia-Silva et al., 2018). See Appendix C.

#### Adherence to LTGO Intervention

The interventionist (BW) kept an attendance record for each of the 12-weekly supervised LTGO exercise and 8-behavior coaching sessions per participant during phase 1, and attendance to the 3-monthly intervention phone calls assessed at the end of phase 2. The degree of adherence was determined by summing the number of weekly supervised exercise sessions and behavior control topics attended during phase 1, and the number of monthly phone calls completed during phase 2. The higher the sum, the greater the adherence.

#### Semi-Structured Interview

Using an interview guide, research assistants (KJ, ET, SJ) who were not involved in the development or delivery of the LTGO intervention, conducted phone interviews with each LTR in the LTGO group at 3 months (completion of phase 1) and 6 months (completion of phase 2). The interviews lasted 15–20 minutes and were audio-recorded and transcribed verbatim. See Appendix D.

### Analyses

All analyses were descriptive. First, quantitative measures were scored according to published guidelines (citations included under the description of each survey). Summary statistics (means ± *SD*, range and proportions) were computed using SPSS v 29.0.2.0 (IBM Corp, 2023) to calculate measures of central tendency. Next, the directed approach to qualitative content analysis (Krippendorff, 2004) was used to systematically code and interpret the interview transcripts using an *a priori* coding scheme based on the major topics that were addressed in the interview scripts. The interview text was segmented into units of analysis (quotes) and tagged with meaningful codes (Hsieh & Shannon, 2005). Two authors (KJ and either ET or SJ) coded the transcripts independently, then compared codes, and reconciled any differences by consensus to create a merged codebook under the direction of a qualitative researcher. Every 5th interview was reviewed to ensure coding consistency. Finally, we looked for patterns and relationships by integrating findings from the SSI and pertinent survey measures that were likely to influence participants’ experiences with the LTGO intervention.

## Results

### Sample Characteristics

The sample included 38 of the 44 LTR who completed an SSI. The mean age was 57.9 years (SD= 11.9) and 34 (82%) of participants were White; 21 (55%) were male. Of all the participants, 20 (52%) reported receiving some college education. Most were married or living with a partner 27 (71%), unemployed or disabled 33 (86%), with 32 (84%) reporting incomes that met their needs.

### Survey Findings

ITFQ scores ranged from 9 to 21 with a mean of 12.05 (±3.1). TUQ scores ranged from 4 to 8.7 with a mean of 6.43 (±0.54). SERE scores ranged from 24.44 to 99.44 with a mean of 66.02 (± 21.72). In phase 1 of the study, participants demonstrated an average adherence of 9.45 out of 12 (79%) weekly exercise training sessions completed and 7.45 of the 8 (93%) behavioral coaching sessions completed. During phase 2, participants completed a mean of 2.23 of the 3 (74%) monthly phone calls. See Table 2.

### Interview Findings

**FIGURES 1-7:**
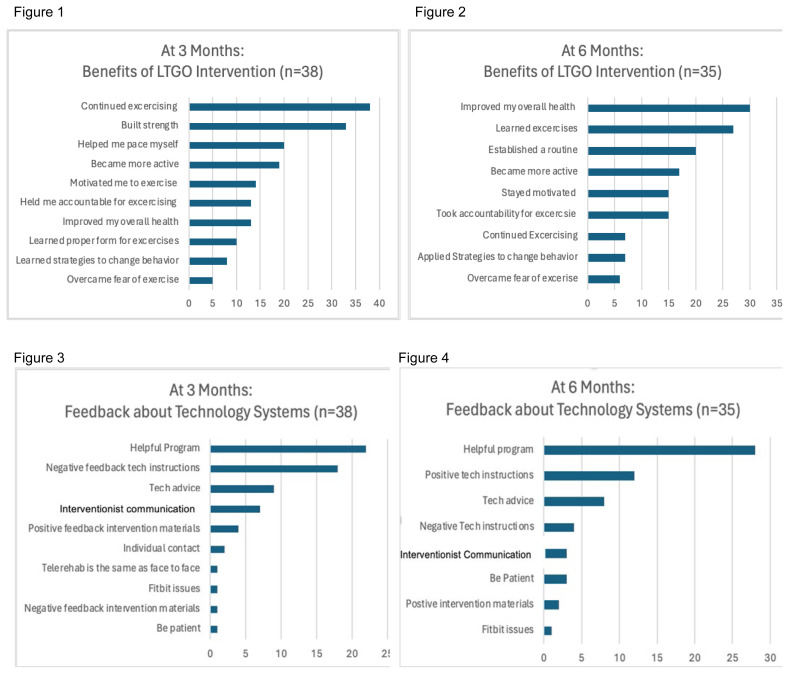

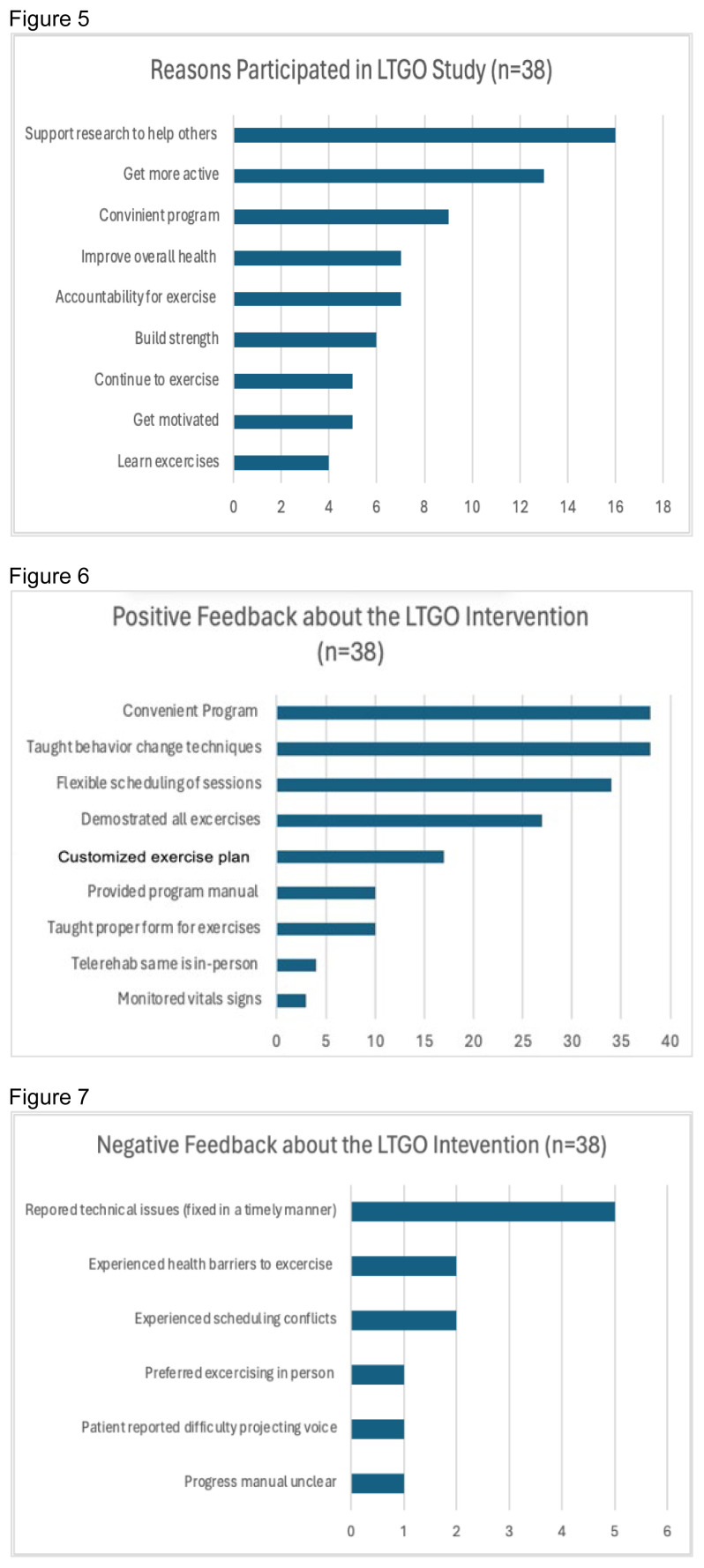
Number of Participants Endorsing Themes at 3 and 6 month Interviews

Figures 1–7 show results of the SSI interviews. At 3 months (Figure 1), participants reported benefits such as motivation to continue exercising and help building strength, structure, and becoming more physically active. At 6 months (Figure 2), responses indicated the primary benefits of the LTGO intervention were improvements in health, ability to learn the exercises, build strength, and establish an exercise routine. At 3 months (Figure 3), when participants were asked to provide feedback about the technology systems, 22 participants stated the system was helpful, but 18 told the interventionist about difficulties they had with the technology at the start of the intervention due to limited clarity or instruction. By 6 months (Figure 4), 28 participants said that the program was helpful; 12 participants expressed positive feedback related to technology, and only 4 expressed negative feedback related to technology.

Responses regarding reasons for participation, positive feedback, and negative feedback at 3 and 6 months were consolidated due to the lack of significant variation between the time points. The most common reasons for participating in the LTGO study were to improve their overall health, convenience of the program, to get more active, and to support research to help others (Figure 5). All participants mentioned the program’s convenience, individualized supervision, and behavior change techniques as positive aspects of the intervention (Figure 6). Despite the video conference format, all participants reported that they were able to communicate adequately with the interventionist during the exercise sessions, except for three who experienced health challenges, scheduling conflicts, and poor voice projection. The five participants who reported technical issues admitted that these were fixed promptly (Figure 7). The critiques of the intervention were a lack of clarity in the exercise manual and a preference for in-person exercise.

### Integration of Multi-Method Findings

Due to the overall homogeneity of findings with generally high scores on the quantitative measures and positive responses on the SSI within the sample, we focused on patterns in the qualitative responses for the subsample of participants (n = 4) who scored below 6 on the TUQ total (using the median as the cut off) and less than 3 on selected TUQ items indicating they may have had technology experience issues. After selecting these outlying cases, we created a joint display (Table 3) of the TUQ total score and TUQ items alongside relevant participant experiences reported during the interviews to gain a deeper understanding of possible explanations of these outlying cases.

Of the participants selected who indicated significant difficulty or resistance to the use of technology in the study, participant 1045, rated the highest number of TUQ items below 3 and had one of the lowest TUQ total scores. The participant’s responses to the TUQ and SSI items suggest that they may have faced widespread challenges with the telerehabilitation systems which may have impacted their perception of the intervention. According to the participant’s low rating of the specific TUQ item “ACCESS” they did not believe that telerehabilitation improved their access to healthcare. This perception seems to stem from issues while using 2-way video conferencing systems, despite their familiarity with information technology indicated by their low ITFQ score. This participant also struggled with communicating with the interventionist, as they rated items related to their ability to “HEAR” and “TALK” with the interventionist low, which could have hindered their ability to fully engage in the intervention. Additionally, their TUQ responses indicated that they believed that adequate instructions were not provided when faced with technological errors, which could have worsened their perception of the usability of the technology used in the intervention. The participant reinforced the frustrations they faced due to technology in their SSI responses as they mentioned a preference for in-person interactions despite believing that the virtual meetings were the same as meeting in-person. Additionally, the participant was the one who mentioned that technology “ERRORS” they faced discouraged them from participating in this study again.

Participant 1100 rated items related to “SAME” and “ERROR” of the TUQ low, indicating that they did not feel that telerehabilitation visits were the same as in-person visits and when faced with errors they did not have clear directions to troubleshoot them. Despite the patient’s low TUQ score, in the SSI they discussed the associated technological processes as being easy and the technology used in the intervention as helpful. As this patient’s ITFQ score was also low (higher familiarity), it is possible that the previous familiarity with technology may have lowered the overall impact of frustrations related to troubleshooting technological errors with the LTGO intervention. As the patient identified that they would participate in this study again, it appears that the difficulty they faced did not significantly affect their acceptance of the telerehabilitation intervention.

Participant 1256 also rated item “ERROR” low indicating that they did not feel that there was a clear direction to troubleshoot technological issues. Additionally, by rating item “SAME” low, this patient indicated that they did not experience the similar benefits from virtual meetings as with in-person visits. However, like Participant 1045, they also expressed a similar, limited level of frustration related to technology and stated that the program was still helpful. Although they expressed a desire for the technology to be easier to set-up or program, they would still participate in the study again. As this patient’s adherence was within an acceptable range, it appears that none of the negative perceptions of the usability of telerehabilitation significantly affected their engagement in the study.

Participant 1270 had a higher ITFQ score, indicating decreased familiarity with information technology, which may explain their lower TUQ score. This patient did not believe they could become productive using this telerehabilitation system quickly and conveyed that it was difficult to learn to use the telerehabilitation system. Additionally, this patient rated TUQ item related to “MISTAKE” low, indicating that they could not recover easily and quickly when using the telerehabilitation system. Despite this patient having the lowest TUQ score, they did not have technology related complaints when answering the relevant SSI questions stating that, “... overall, the intervention and associated technology was helpful and that they would participate in this study again.” This suggests that most of the struggle and any negative perceptions of telerehabilitation from this patient stemmed from an initial lack of familiarity with information technology and having to learn how to use technology for the study rather than a lack of acceptability of telerehabilitation systems. However, the issues that this patient reported with technology indicated by their TUQ and IFTQ scores could have resulted in their low adherence to intervention sessions.

## Discussion

The LTGO telerehabilitation intervention was intended to improve the physical activity and physical function of LTR by combining individualized supervised exercise training with behavioral coaching. This report described the personal experiences of LTR randomized to the LTGO intervention and the factors that influenced the participants’ subjective experience with a telerehabilitation behavioral intervention. The findings highlight insights into how participants engaged with the technology used in this intervention, and how various factors may have influenced their overall perceptions.

The mean ITFQ of 12, out of a possible score of 21, indicated that most participants had a moderate level of familiarity with the technologies used in the LTGO intervention, such as video conferencing platforms. Since higher ITFQ scores indicate less familiarity with information technology, the slightly higher mean for the ITFQ may indicate some barriers related to confidence in using technology or ease of use which could affect participants’ overall engagement in the study or efficacy of the intervention.

The high mean for the TUQ of 6.43, out of a scale of 7, indicates that the telerehabilitation system was generally acceptable for the participants. This high score suggests there was a positive perception of the technology associated with this intervention about the six aspects of usability rated in this measure. However, it is noteworthy that 4 of the 7 participants with the lowest TUQ scores also had ITFQ scores above the mean, indicating worse pre-intervention familiarity with information technology, suggesting that lower familiarity with technology may have contributed to more negative perception of the usability of telerehabilitation systems. A mean score of 66.02 (+ 21.72) out of 100 for the SERE indicates a moderately high level of self-efficacy among participants and self-perception that participants have the confidence to complete an exercise routine regularly. This high level of self-efficacy is reflected in the generally higher rates of adherence to the LTGO intervention sessions in phase 1 and 2 over a 6-month period.

Adherence to the weekly exercise training sessions in phase 1 was high at 79%. This adherence rate reflects consistent participation but suggests that some barriers prevented 100% adherence. Barriers to adherence reportedly stemmed from personal scheduling issues, post-lung transplant health complications, and problems with technology. Despite these factors, the high rate of adherence suggests that participants were still committed to participating in the intervention. The high rate of adherence for the behavioral coaching sessions (93%) in comparison to the weekly exercise training sessions (79%) and monthly phone calls (74%) suggests that participants may have found these coaching sessions particularly valuable as education was provided on SMART goal setting, time management, how to deal with setbacks or barriers and more topics to facilitate changes in health behavior. Furthermore, participants may have seen these change techniques as easier to perform because they involved fewer logistical and technological barriers.

The adherence rates for the monthly phone calls in phase 2 (74%) were also high, but slightly lower than adherence to the phase 1 weekly exercise training and behavioral coaching sessions. This drop in adherence could be attributed to the change in the intervention delivery mode from face-to-face video calls in phase 1 to phone calls in phase 2. During the SSI at 3 months, many participants reported that accountability and motivation to exercise were significant benefits of this intervention. In contrast, the phone call format in Phase 2 may have been less engaging, potentially reducing the sense of accountability felt by participants, due to the decreased frequency of communication and more impersonal nature of the phone calls. Other barriers like personal scheduling conflicts or technological issues may have further impacted adherence to these sessions. Despite these barriers, the high percentage of adherence indicates that participants valued these sessions, even as the communication format transitioned to phone calls. This continued engagement speaks to the general acceptability of the telerehabilitation format utilized in the LTGO intervention.

However, the negative feedback at 3 months related to technology, obtained from the SSI, suggests that participants felt frustrations related to the use of technology in this intervention which could affect their perception of telerehabilitation or the efficacy of the LTGO intervention. These struggles were not always rooted in an early lack of familiarity with telerehabilitation systems or a lack of familiarity with technology in general. The decrease in negative feedback related to technology from 3 months to 6 months indicates that as participants were given proper instructions and support to become more familiar with the telerehabilitation systems utilized in the intervention, the participants’ perception of the acceptably of telerehabilitation systems may have positively changed by the end of the intervention.

The shift in feedback from short-term benefits, such as being more active at 3 months, to longer-term benefits, like improvements in overall health and establishment of an exercise routine at 6 months, suggests that the LGTO intervention facilitated both immediate and long-term gains. It appears that this intervention has effectively prepared participants to create sustainable lifestyle changes as participants incorporate the exercise related behaviors that they learned during the intervention into their daily lives. Additionally, all participants highlighted the program’s convenience, enabled by its virtual format, as a positive aspect of this intervention. This convenience may also be a reason for the increased acceptability of the telerehabilitation intervention, despite initial challenges.

The findings of the multi-methods inquiry may indicate that even negative experiences with technology during this telerehabilitation intervention may not necessarily deter participation in such programs. Despite having TUQ scores significantly lower than the mean, indicating lower usability of the technology used in the LTGO intervention, 3 of 4 participants selected for the multi-method inquiry expressed that they would participate in this intervention again. This suggests that negative perceptions of the usability of technology did not generally negatively impact the acceptability of this telerehabilitation intervention. However, these participants’ responses to various measures reveal areas of concern where the LTGO intervention may be improved. For instance, improving the participants’ ability to hear and see the interventionist during the intervention and teaching participants how to troubleshoot the associated telerehabilitation systems in more detail. Improving these factors could further address usability and acceptability issues and improve the intervention for future participants as this intervention is more widely implemented.

This study revealed areas such as lack of prior exposure to information technology in which the LTGO study could be revised to increase the general acceptability of the telerehabilitation intervention proved by the LTGO study. In the future, this inquiry should be repeated while relating indicators of participant experience to intervention outcomes to identify specific areas in which the intervention can be improved. Additionally, measures like the ITFQ and TUQ could be used to identify participants who may benefit from additional support in setting up and using the technology associated with various aspects of the intervention, such as additional education or video tutorials.

### Limitations

While this study provides valuable insights into participants’ perceptions of the LTGO intervention, it is important to consider possible limitations. Racial demographic representation was low with 82% of participants being white. However, the sample characteristics of this study were comparable to the general population of lung transplant recipients in the U.S. (Valapour et al., 2023). Another potential limitation was possible recall bias, as the interviews were conducted at the end of phase 1 and phase 2 which did not capture the participants’ experiences and perceptions in real time, thus reducing its validity, especially when comparing responses between time points (Crafoord et al., 2020). However, the interviews were conducted at the same time points across the sample (3 and 6 months) which may mitigate the potential bias and enhance the validity through integration of findings from multi-methods. Alternatively, the post-intervention timing of the interviews may have allowed participants time to identify the longer-term impacts of the LTGO intervention that may have otherwise been overlooked.

## Conclusion

While telerehabilitation-mediated self-management is not always superior to usual care, participant responses did not report any negative effects, suggesting that telerehabilitation is a convenient and safe option for the delivery of self-management support, particularly in conditions such as heart failure and type 2 diabetes, where the evidence base is more developed (Hanlon et.al., 2017). Participants reported positive subjective experiences in the LTGO telerehabilitation intervention, suggesting that this method of long-term care delivery may be particularly practical and valuable for lung transplant recipients. The high level of adherence and willingness to participate again in this intervention, despite frustrations with the associated technology in outlying cases, underscore the acceptability of this model, especially due to its convenience compared to in-person PR. Furthermore, the availability of the LTGO study protocol (Vendetti, et al., 2023), exercise progression protocol (Hergendroeder, et al., 2023), procedures for conducting remote assessments of physical function and physical activity (Moon et al., 2021), and the script and coding scheme for the SSI presented here to enhance reproducibility. Larger-scale trials of telerehabilitation-supported self-management, based on explicit self-management and behavior change theory, are needed before the extent to which telerehabilitation technologies may be harnessed to promote better outcomes after lung transplantation.

## Figures and Tables

**Table 1:** Sample Characteristics (n=38)

Age, M (SD)	57.9 (11.8)
Male (n/%)	21 (55)
Race, White (n/%)	34 (89)
Black	2 (5)
Asian	1 (3)
Married or living with partner (n/%)	27 (71)
Education, any college (n/%)	20 (52)
Unemployed or disabled (n/%)	33 (86)
Income meets needs (n/%)	32 (84)
Double transplant (n/%)	38 (100)

**Table 2:** Findings of Survey Measures (n=38)

Measure	Mean	SD	Range
ITFQ score	12.05	3.1	9–21
TUQ score	6.43	0.54	4.8–7
SERE score	66.03	21.72	24.44–99.44
Exercise training sessions completed (Phase 1)	9.45	3.95	0–12
Behavioral coaching sessions completed (Phase 1)	7.45	1.48	0–8
Phone calls completed (Phase 2)	2.23	1.25	0–3

ITFQ = The Information Technology Familiarity Questionnaire; TUQ = Telerehabilitation Usability Questionnaire; SERE= Self-Efficacy to Regulate Exercise

**Table 3:** Joint Display for Sub-Sample (n=4): TUQ Total Score, TUQ Item, and Coded Semi-Structured Interview Responses by Participant ID

Participant ID	Total TUQ	TUQ item	Coded Interview Responses
1045	4.81	ACCESS	3. Tech issues
		TALK	4b. Face to Face, Individual
		HEAR	Contact, Positive tech
		ERROR	Instructions
			5b. Prefer in person
1100	5.81	SAME	3. Helpful
		ERROR	4b. Helpful, Positive Intervention, Positive tech
			5b. Would participate again
1256	5.86	SAME	3. Helpful
		ERROR	4b. Tech advice
			5b. Would participate again
1270	4.65	USE	3. Helpful
		LEARN	4b. Helpful
		RECOVER	5b. Would Participate again

TUQ = Telerehabilitation Usability Questionnaire

*Note*: ID 1045 and 1100 had low IFTQ familiarity scores; ID 1270 reported low intervention adherence

## Appendix A Information Technology Familiarity Questionnaire (ITFQ)

**Table:**

Circle the number that corresponds most closely to your use of a computer or smart phone to access the internet.	
Rating: 1= Daily use for this activity 2= Seldom use for this activity 3= Never use for this activity	
Question	Rating
1. I use my computer or smart phone to send and receive email.	3 / 2 / 1
2. I use my computer or smart phone to obtain information on a wide range of topics.	3 / 2 / 1
3. I download applications from the internet to my computer of smart phone.	3 / 2 / 1
4. I use my computer or smart phone to shop, manage my calendar and/or make travel arrangements.	3 / 2 / 1
5. I use my computer or smart phone to bank and pay my bills.	3 / 2 / 1
6. I use my computer or smart phone for social networking.	3 / 2 / 1
7. I use my computer or smart phone for movies, videos, podcasts, music, or sharing photos/images.	3 / 2 / 1
8. I use other forms of electronic technology (eBooks, Kindle, NookBook) or tablets (iPad, etc).	3 / 2 / 1

## Appendix B Telehealth Usability Questionnaire (TUQ)

**Table:**

	N/A		1	2	3	4	5	6	7	
*1. Telehealth improves my access to healthcare services. (ACCESS)	□	Disagree	□	□	□	□	□	□	□	Agree
2. Telehealth saves me time traveling to a hospital or specialist clinic.	□	Disagree	□	□	□	□	□	□	□	Agree
3. Telehealth provides for my healthcare need.	□	Disagree	□	□	□	□	□	□	□	Agree
*4. It was simple to use this system. (USE)	□	Disagree	□	□	□	□	□	□	□	Agree
*5. It was easy to learn to use the system. (LEARN)	□	Disagree	□	□	□	□	□	□	□	Agree
6. I believe I could become productive quickly using this system.	□	Disagree	□	□	□	□	□	□	□	Agree
7. The way I interact with this system is pleasant.	□	Disagree	□	□	□	□	□	□	□	Agree
8. I like using the system.	□	Disagree	□	□	□	□	□	□	□	Agree
9. The system is simple and easy to understand.	□	Disagree	□	□	□	□	□	□	□	Agree
10. This system is able to do everything I would want it to be able to.	□	Disagree	□	□	□	□	□	□	□	Agree
*11. I can easily talk to the clinician using the telehealth system. (TALK)	□	Disagree	□	□	□	□	□	□	□	Agree
*12. I can hear the clinician clearly using the telehealth system. (HEAR)	□	Disagree	□	□	□	□	□	□	□	Agree
13. I felt I was able to express myself effectively.	□	Disagree	□	□	□	□	□	□	□	Agree
14. Using the telehealth system, I can see the clinician as well as if we met in person.	□	Disagree	□	□	□	□	□	□	□	Agree
*15. I think the visits provided over the telehealth system are the same as in-person (SAME)	□	Disagree	□	□	□	□	□	□	□	Agree
*16. Whenever I made mistake using the system, I could recover easily and quickly. (MISTAKE)	□	Disagree	□	□	□	□	□	□	□	Agree
*17. The system gave error messages that clearly told me how to fix problems. (ERROR)	□	Disagree	□	□	□	□	□	□	□	Agree
18. I feel comfortable communicating with the clinician using the telehealth system.	□	Disagree	□	□	□	□	□	□	□	Agree
19. Telehealth is an acceptable way to receive healthcare services.	□	Disagree	□	□	□	□	□	□	□	Agree
20. I would use telehealth services again.	□	Disagree	□	□	□	□	□	□	□	Agree
21. Overall, I am satisfied with this telehealth system.	□	Disagree	□	□	□	□	□	□	□	Agree

*Note*:

* items 1,4,5,11,12,15,16,17 selected for inclusion in the multi-method analyses (if rated < 3); Key words were used to label the item succinctly.

## Appendix C Self-Efficacy to Regulate Exercise (SERE)

**Table:**

Exercise Self-Efficacy: Self-Efficacy to Regulate Exercise (SERE)										

A number of situations can make it hard to stick to an exercise routine. Please rate how confident you are that you will be able to perform your exercise routine regularly (three or more times a week) under each of the following situations.										

Rate your degree of confidence by recording a number from 0 to 100 using the scale given below:										

*0*	*10*	*20*	*30*	*40*	*50*	*60*	*70*	*80*	*90*	*100*
*Cannot do at all*					*Moderately can do*					*Highly certain can do*

										Confidence (0–100)

1.	When I am feeling tired									

2.	When I am feeling under pressure from work (work is not limited to employment and can mean whatever it means to you)									

3.	During bad weather									

4.	After recovering from an injury that caused me to stop exercising									

5.	During or after experiencing a personal problem									

6.	When I am feeling depressed									

7.	When I am feeling anxious									

8.	After recovering from an illness that caused me to stop exercising									

9.	When I feel physical discomfort when I exercise									

10.	After a vacation (if you were to go on vacation)									

11.	When I have too much work to do at home (whether working on a job or at home)									

12.	When visitors are present									

13.	When there are other interesting things to do									

14.	If I don’t reach my exercise goals									

15.	Without support from my family or friends									

16.	During a vacation (if you were to go on vacation)									

17.	When I have other time commitments									

18.	After experiencing family problems									

## Appendix D Semi-Structured Interview Script

**Table:**

1. Tell me why you decided to participate in the 6-month exercise program using Two-way communication system:
2. Describe ways the 6-month exercise program using Two-way communication system was helpful so far?
*3. Describe ways the 6-month exercise program using Two-way communication system was NOT helpful so far?
*4. What suggestions do you have for improving this experience for other patients who have undergone lung transplantation in relation to: 4a. Ease of learning the exercises: 4b. Ease of using the system: 4c. Ease of communicating with the research team
*5. If a home exercise program using two-way communication system becomes available to you again, would you like to participate? (Yes or No) 5a. Please explain the reasons why you WOULD participate again. 5b. Please explain the reasons why you WOULD NOT participate again.
6. Would you recommend this intervention to other lung transplant recipients?
7. Do you have any other information that you would like to share with us about being part of this research study?

*Note*:

* SSI questions 3, 4 and 5 selected for inclusion in the multi-method analyses

## Appendix E Code Book for Semi-Structured Interviews

**Table:**

Codes	Root Words	Definition
ACC	Accountability	Having someone hold them accountable was positive
ACTIVE	Active	Get Active again, help them get moving
BEH_POS	Behavior Positive	Found benefit in the behavior models
COMM_ADVICE	Communication Advice	Advice to make communicating with the research team easier
COMM_ISSUES	Communication Issues	There were issues with communication
COMM_POS	Positive communicating w/Interventionist	Interventionist was good at communicating
CONT_EX	Continue exercising	The program helped them to continue exercising
FACE_TO_FACE	Face to face	Patient thinks the sessions are the same as face to face visits or feels as if they were face to face
CONVEN	Convenient	The program was good because of its convenience
ENCOURAGE	Encouragement	Interventionist’s encouragement helped them
EX_ADVICE	Exercise Program Advice	Add more exercises or add more contact/sessions
EX_DIFF	Exercising difficulties	Patient mentions there were barriers to their exercises (health, injuries, hospitalizations, etc.)
EX_INS_NEG	Negative exercise explanation	There were difficulties understanding exercises or confusion with exercises
EX_INS_POS	Positive exercise explanation	The exercises were easy to learn and to understand
FORM	Form	Help to teach form or adjust form
FITBIT_ISSUES	Fitbit Issues	There were issues with the Fitbit discussed
HEALTH	Improve overall health	The program helped to improve overall health
HELP_OTH	Help others/research	They were involved in the program because they wanted to help others
HELPFUL	Helpful	They were involved in the program because they wanted to help others
INDIVIDUAL	Individual contact	Individual, 1 on 1, work with the interventionist, instant feedback
INTMAT_NEG	Negative intervention materials feedback	There was negative feedback on the intervention materials
INTMAT_POS	Positive intervention materials feedback	There was positive feedback on the intervention materials
LEARN	Learn exercises	Patient feels like they learned new exercises and can use it moving forward
MONITOR_VIT	Monitor vitals	Helped to more regularly check on vitals
MOTIV	Motivation	A patient discusses motivation through the program
OVER_FEAR	Motivation	Patient overcomes fear of exercising
PACE_POS	Good program speed, gradual progressions	The program is run at a good pace.
PACE_NEG	Negative feedback on program speed	The program speed was not good for the patient
PATIENT	Be patient	The patient appreciated Interventionist’s patience with them
PREF_INEPERS	Physically in person	Prefer to be physically in person for assistance
STRENGTH	Build strength	Mentioning the program helps you build strength
STRUCTURE_POS	Structure positive	Having structure is a benefit
TECH_ADVICE	Tech advice	Tech advice to make the set-up or program easier
TECH_INS_NEG	Negative tech (Zoom) instructions	Discusses difficulties that they had with technology due to lack of clarity or instruction
TECH_INS_POS	Positive tech (Zoom) instructions	Discussed the tech process as being easy
TECH_ISSUES	Tech issues	A patient describes having technical issues
TIME_FLEX_POS	Flexibility-time	The time commitment was good due to Interventionist’s flexibility
TIME_ISSUES	Time constraint issues	The time constraint was a problem
VOICE	Difficulty projecting voice	Patient difficulties with communicating due to inability to project voice post-transplant
